# P-1170. Impact of Healthcare-associated Respiratory Virus Infections in Children

**DOI:** 10.1093/ofid/ofae631.1356

**Published:** 2025-01-29

**Authors:** Aki Miyashita, Yuho Horikoshi, Hanako Funakoshi, Meiwa Shibata, Keiko Nakamura, Shigeko Miyokawa

**Affiliations:** Division of Infectious Diseases, Department of Pediatrics, Tokyo Metropolitan Children’s Medical Center, Tokyo, Japan., Fuchu, Tokyo, Japan; Division of Infectious Diseases, Department of Pediatrics, Tokyo Metropolitan Children's Medical Center, Fuchu, Tokyo, Japan; Division of Infectious Diseases, Department of Pediatrics, Tokyo Metropolitan Children’s Medical Center, Tokyo, Japan., Fuchu, Tokyo, Japan; Division of Infectious Diseases, Department of Pediatrics, Tokyo Metropolitan Children’s Medical Center, Tokyo, Japan., Fuchu, Tokyo, Japan; 2) Department of Nursing, Tokyo Metropolitan Children’s Medical Center, Tokyo, Japan, Fuchu, Tokyo, Japan; 3) Department of Nursing, Tokyo Metropolitan Neurological Hospital, Tokyo, Japan, Fuchu, Tokyo, Japan

## Abstract

**Background:**

Respiratory viruses can spread within healthcare facilities despite stringent infection prevention measures. Although healthcare-associated respiratory virus infections (HARVI) pose a significant risk to children, pediatric HARVI are understudied. The present study aimed to evaluate the impact of HARVI on children following the SARS-CoV-2 pandemic at a tertiary children’s hospital.

**Methods:**

This retrospective study enrolled pediatric inpatients aged ≤18 years who were hospitalized at Tokyo Metropolitan Children's Medical Center between April 2020 and March 2024. HARVI was defined as a viral infection with a new onset of respiratory symptoms past the incubation period following hospitalization. The causative virus was detected using multiplex PCR (FilmArray Respiratory Panel 2.1, BioFire). Patients with other infections were excluded. The primary outcome was the 30-day mortality rate. The secondary outcomes included PICU admission, escalation of respiratory support, and the postponement of discharge, planned surgery or chemotherapy.

**Results:**

In total, 265 patients with HARVI were enrolled. Male patients comprised 31% of the cohort, and the median age was 31 (IQR [interquartile range]: 12-77) months. Underlying neurologic, cardiac, and congenital or genetic disorders had in 42%, 28%, and 24% of the cohort, respectively. The respiratory viruses detected were the rhinovirus/enterovirus (44%), parainfluenza virus type 3 (15%), and HKU1, NL63, 229E and OC43 coronaviruses (8%). The 30-day mortality rate was 1% in three patients with parainfluenza virus type 3, RSV, and SARS-CoV-2. The PICU admission rate was 2%. The rate of respiratory support escalation was 17%. Postponement of discharge was required in 7% of the cohort for a median period of seven (IQR: 5-8) days. Postponement of surgery was required in 3% of the cohort for a median period of 46 (IQR: 21-82) days. There was no postponement of chemotherapy.

**Conclusion:**

Pediatric HARVI was associated with mortality and additional medical care. Therefore, strict infection prevention measures are essential.

**Disclosures:**

**Hanako Funakoshi, MD**, Pfizer Japan: Husband is an employee.

